# Adaptive designs were primarily used but inadequately reported in early phase drug trials

**DOI:** 10.1186/s12874-024-02256-9

**Published:** 2024-06-05

**Authors:** Yuning Wang, Minghong Yao, Jiali Liu, Yanmei Liu, Yu Ma, Xiaochao Luo, Fan Mei, Hunong Xiang, Kang Zou, Ling Li, Xin Sun

**Affiliations:** 1 Institute of Integrated Traditional Chinese and Western Medicine, West China Hospital, Chinese Evidence-based Medicine Center and Chinese Cochrane Center, Sichuan University, Chengdu, 610041 China; 2 NMPA Key Laboratory for Real World Data Research and Evaluation in Hainan, Chengdu, 610041 China; 3 China Sichuan Center of Technology Innovation for Real World Data, Chengdu, 610041 China

**Keywords:** Adaptive design, Randomized control trial, Reporting quality, Group sequential design

## Abstract

**Background:**

Faced with the high cost and limited efficiency of classical randomized controlled trials, researchers are increasingly applying adaptive designs to speed up the development of new drugs. However, the application of adaptive design to drug randomized controlled trials (RCTs) and whether the reporting is adequate are unclear. Thus, this study aimed to summarize the epidemiological characteristics of the relevant trials and assess their reporting quality by the Adaptive designs CONSORT Extension (ACE) checklist.

**Methods:**

We searched MEDLINE, EMBASE, Cochrane Central Register of Controlled Trials (CENTRAL) and ClinicalTrials.gov from inception to January 2020. We included drug RCTs that explicitly claimed to be adaptive trials or used any type of adaptative design. We extracted the epidemiological characteristics of included studies to summarize their adaptive design application. We assessed the reporting quality of the trials by Adaptive designs CONSORT Extension (ACE) checklist. Univariable and multivariable linear regression models were used to the association of four prespecified factors with the quality of reporting.

**Results:**

Our survey included 108 adaptive trials. We found that adaptive design has been increasingly applied over the years, and was commonly used in phase II trials (*n* = 45, 41.7%). The primary reasons for using adaptive design were to speed the trial and facilitate decision-making (*n* = 24, 22.2%), maximize the benefit of participants (*n* = 21, 19.4%), and reduce the total sample size (*n* = 15, 13.9%). Group sequential design (*n* = 63, 58.3%) was the most frequently applied method, followed by adaptive randomization design (*n* = 26, 24.1%), and adaptive dose-finding design (*n* = 24, 22.2%). The proportion of adherence to the ACE checklist of 26 topics ranged from 7.4 to 99.1%, with eight topics being adequately reported (i.e., level of adherence ≥ 80%), and eight others being poorly reported (i.e., level of adherence ≤ 30%). In addition, among the seven items specific for adaptive trials, three were poorly reported: accessibility to statistical analysis plan (*n* = 8, 7.4%), measures for confidentiality (*n* = 14, 13.0%), and assessments of similarity between interim stages (*n* = 25, 23.1%). The mean score of the ACE checklist was 13.9 (standard deviation [SD], 3.5) out of 26. According to our multivariable regression analysis, later published trials (estimated *β* = 0.14, *p* < 0.01) and the multicenter trials (estimated *β* = 2.22, *p* < 0.01) were associated with better reporting.

**Conclusion:**

Adaptive design has shown an increasing use over the years, and was primarily applied to early phase drug trials. However, the reporting quality of adaptive trials is suboptimal, and substantial efforts are needed to improve the reporting.

**Supplementary Information:**

The online version contains supplementary material available at 10.1186/s12874-024-02256-9.

## Background

Randomized controlled trials (RCTs) are considered the “gold standard” for assessing the clinical efficacy of interventions. However, the high cost and limited efficiency associated with classical RCTs [1, 2] have exposed the need for more efficient designs. Adaptive design, characterized by its flexibility and efficiency, allows for timely decision making based on accumulating trial data [3, 4], such as stopping trials early [5], allocating more participants to better groups [6], or dropping inefficient arms [7]. The advantages of adaptive design in reducing research time [8, 9], saving sample size [10, 11], and improving success rates [12, 13] have prompted many researchers to incorporate it into the new drug development process.

Reviews [14–17] have specifically focused on the application and reporting of adaptive trials. One review [14] including adaptive trials other than phase I and seamless phase I/II trials, found that seamless phase II/III trials were the most frequently used type, and that many researchers had failed to adequately report dependent monitoring committees (DMCs) and blinded interim analyses. Another literature survey [17], including phase II, phase III, and phase II/III adaptive trials in oncology, found that adaptive design was commonly applied in phase III trials and that the reporting of adaptive design-related methods was inadequate. A review [15] summarizing features of 60 adaptive trials with specific methodology types showed that the statistical method descriptions were poor. A systematic review [16] assessing the reporting compliance of group sequential RCTs by Consolidated Standards of Reporting Trials (CONSORT) 2010 checklist revealed a lack of accessibility to protocols for details. However, these studies had important limitations for addressing the application and reporting of adaptive trials. First, the included adaptive trials were restricted to specific clinical phases and areas of disease. Second, the studies were focused on identifying deficiencies on specific aspects of interest (e.g., statistical methods). Third, none of the studies focused on drug trials. Thus, the findings of those studies were not comprehensive and may not be generalizable to other adaptive design types.

The Adaptive designs CONSORT Extension (ACE) statement, a reporting guidance for adaptive trials, was developed in 2020 to advise clinical researchers on how to report details of the adaptive design [18]; this statement is also considered a valid tool to evaluate the reporting quality of adaptive trials. Our study aimed to retrieve adaptive drug RCTs in all phases and disease areas to systematically investigate the overall application of adaptive design to drug RCTs, comprehensively identify gaps in reporting, and investigate the extent to which adaptive design information was reported before the publication of the ACE checklist, to provide evidence leading to directional improvements and advocacy for adequate reporting in the future.

## Materials and methods

### Eligibility criteria

We selected studies according to the following criteria: (1) RCTs explicitly stating to be adaptive trials or applying any type of adaptive design; (2) RCTs assessing efficacy or safety of drugs; and, (3) RCTs published in English journals. We excluded: (1) re-published studies; (2) protocols, abstracts, or re-analyses of adaptive trials; and, (3) incomplete trials.

### Search strategy and screening

We searched EMBASE, MEDLINE, Cochrane Central Register of Controlled Trials (CENTRAL), and ClinicalTrials.gov databases from inception to January 2020. We used both subject headings and free-text terms related to adaptive clinical trials to identify relevant studies (See Appendix 1 for the search strategy).

### Data extraction

We generated a data extraction table to record the following information: first author, publication year, journal (quantile 1 defined by Journal Citation Reports [JCR], others), reasons for utilizing adaptive designs, trial center (multicenter, single-center), whether a trial was international or not, trial clinical phase, adaptive design type, area of disease, type of control (active, non-active, both), type of primary outcome, expected sample size, randomized sample size, and funding source (government, private for-profit, private not-for-profit, not funded, or unclear).

We extracted primary outcome according to the following strategy: (1) if a trial specified primary outcome(s), we selected it or the first one as the primary outcome; (2) if a trial did not specify primary outcomes, we selected the first one reported in the results. Further, we classified these selected primary outcomes into two types: clinical outcomes (clinically meaningful endpoints that directly measured how patients feel, functions, or survives) or surrogate endpoints (laboratory measures or physical signs intended to be substitutes for clinically meaningful endpoints) [19].

Based on the literature [12–14], we classified adaptive designs into 10 types: group sequential, adaptive dose-finding, adaptive randomization, sample size re-estimation, adaptive hypothesis, biomarker adaptive, seamless, pick the winner/drop the loser, adaptive treatment-switching, and multiple adaptive designs. We identified and extracted the adaptive design types as planned, regardless of whether they were implemented, which avoided the omission of any types.

### Reporting quality assessment

ACE checklist, a specific CONSORT extension to adaptive trials, provided essential reporting requirements to enhance transparency and improve reporting. Hence, we assessed the reporting quality of the included studies by the ACE checklist. First, we evaluated the adaptive RCTs’ compliance for 26 topics of ACE checklist. Second, we also assessed seven essential items (new items) specific to adaptive trials in the ACE checklist, nine modified items relative to the CONSORT 2010 checklist, and six items with expanded text for adaptive design. The response to each topic/item could be “yes”, “no”, or “not applicable”, indicating compliance with ACE, non-compliance, or not applicable, respectively. Based on previous literature, we selected proportions of adherence ≤ 30% as underreporting [20–22]. Due to the complexity of the adaptive design, we chose a strict threshold of 80% adherence to define good reporting [23, 24]. To quantify the reporting quality, we used a scoring strategy for every topic, assigning 1 point to “yes” or “not applicable” and 0 points to “no” [3, 4, 25], with a total score ranging between 0 and 26.

### Study process

Two-paired method-trained researchers screened abstracts and full texts for eligibility and then independently extracted data from eligible trials using predesigned standardized forms with detailed instructions. Additionally, two researchers trained in the ACE checklist independently assessed the reporting quality of the studies included. Any disagreements were resolved through discussions or after consultation with a third researcher.

### Statistical analysis

We used R (4.2.0) for statistical descriptions and analyses. We summarized epidemiological characteristics and reporting adherence on the basis of the extracted data. We reported frequencies with proportions for categorical data and means with standard deviations (SD) or medians with first and third quartiles for continuous data. We compared characteristics and adherence of reporting between trials from quantile 1 (Q1) of JCR and others to identify any potential differences, using chi-square or Fisher’s exact tests for categorical data and Student’s *t*-tests (if data were normal and variances were homogeneous) or Wilcoxon rank-sum tests for continuous data.

To explore factors associated with the overall reporting scores, we developed univariable and multivariable linear regression models, selecting four factors: publication year (as a continuous variable), trial center type (1 for “multicenter” and 0 for “single-center”), type of outcome (1 for “clinical outcome” and 0 for “surrogate endpoint”), and funding source (1 for “private for-profit” and 0 for “others”). Our aim was to determine whether later published trials or multicenter trials had better reporting quality, possibly due to improved understanding and stringent quality control measures. Additionally, we sought to explore whether the type of outcome and funding source influenced the conduct and reporting of adaptive trials. We tested basic assumptions for the models: whether the residuals followed a normal distribution and whether collinearity among the factors existed (VIF > 10) (α = 0.05).

## Results

### Literature screening results

Our search yielded 4891 records published in English. After removing duplicates, we screened titles and abstracts of 3597 records according to the eligibility criteria. We assessed the eligibility of the 341 selected records by reading their full texts. Finally, we included 108 clinical trials from 107 records (where one record included two trials), in our survey (Fig. S1).

### Epidemiological characteristics of included studies

**Fig. 1:**
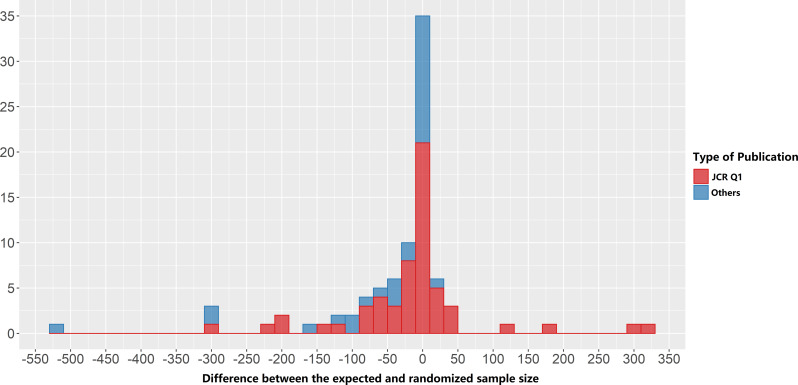
Difference between the expected and randomized sample size of included trials^a^. ^a^Only one difference (-4829) was less than − 550 and was not shown in the figure due to its small size

The use of adaptive design has shown an increasing trend over the years (Figure S2). Group sequential (*n* = 63, 58.3%), adaptive randomization (*n* = 26, 24.1%), adaptive dose-finding (*n* = 24, 22.2%), sample size re-estimation (*n* = 17, 15.7%), and adaptive hypothesis (*n* = 16, 14.8%) designs were common types planned in adaptive trials. In addition, 52 trials (48.1%) were planned to apply multiple types of adaptive design. Adaptive designs were mostly used to speed trials and facilitate decision-making (*n* = 24, 22.2%), maximize the benefit of participants (*n* = 21, 19.4%), and reduce the total sample sizes (*n* = 15, 13.9%). We quantitatively present the total sample size reductions after calculating the difference between the expected and randomized sample sizes (Fig. 1). The range of this difference was between − 4829 and 319, with 51 of the reductions (47.2%) being less than 0 (i.e., reducing the total sample size) and 25 (23.1%) being larger than 0.

Of these trials, 68 (63.0%) were multicenter trials, while only 23 (21.3%) were international trials. Phase II trials (*n* = 45, 41.7%) were the most numerous within the adaptive trials, followed by phase III (*n* = 14, 13.0%) and phase I (*n* = 13, 12.0%) trials. The main area of disease was oncology (*n* = 28, 25.9%). The most used types of control were non-active control (*n* = 52, 48.1%) and active control (*n* = 49, 45.4%). We found 64 trials (59.3%) with selected clinical outcomes as primary outcomes, and others (*n* = 44, 40.7%) with selected surrogate endpoints. The medians of the expected and randomized sample sizes were 162 (first, third quartile: 86, 400) and 124 (69, 290), respectively. Most trials, 66 (61.1%), were funded by private for-profit institutions, 29 (26.9%) by governments, and 27 (25.0%) by private not-for-profit institutions (Table 1).

**Table 1:** Characteristics of included studies

Characteristics	Total (%) (*n* = 108)	JCR Q1 (%) (*n* = 71)	Others (%) (*n* = 37)	*P* value
**Reasons for utilizing adaptive design**				
Speed the trial and facilitate decision-making	24 (22.2)	13(18.3)	11(29.7)	0.27
Maximize the benefit of participants	21 (19.4)	16(22.5)	5(13.5)	0.39
Reduce the total sample sizes	15 (13.9)	10(14.1)	5(13.5)	1
Improve certain aspects of trial	13 (12.0)	9(12.7)	4(10.8)	1
Limited prior clinical experience	4 (3.7)	2(2.8)	2(5.4)	0.89
Recruitment and ethical problems	2 (1.9)	0(0)	2(5.4)	0.12^a^
Reduce cost	1 (0.9)	1(1.4)	0(0)	1^a^
Not specified	28 (25.9)	20(28.2)	8(21.6)	0.61
**Type of adaptive design**				
Group sequential design	63(58.3)	43(60.6)	20(54.1)	0.66
Adaptive randomization	26(24.1)	21(29.6)	5(13.5)	0.11
Adaptive dose finding design	24(22.2)	13(18.3)	11(29.7)	0.27
Sample size re-estimation	17(15.7)	9(12.7)	8(21.6)	0.35
Adaptive hypothesis	16(14.8)	13(18.3)	3(8.1)	0.26
Pick the winner/drop the loser	12(11.1)	10(14.1)	2(5.4)	0.30
Adaptive seamless design	7(6.5)	3(4.2)	4(10.8)	0.36
Biomarker adaptive design	4(3.7)	2(2.8)	2(5.4)	0.89
Adaptive treatment switching design	1(0.9)	1(1.4)	0(0)	1.00^a^
Multiple adaptive design	52(48.1)	35(49.3)	27(73.0)	0.03
**Multicenter trial**	68(63.0)	44(62.0)	24(64.9)	0.93
**International trial**	23(21.3)	20(28.2)	3(8.1)	0.03
**Clinical phase**				0.01^a^
Phase I	13(12.0)	4(5.6)	9(24.3)	
Phase II	45(41.7)	36(50.7)	9(24.3)	
Phase III	14(13.0)	7(9.9)	7(18.9)	
Phase IV	2(1.9)	2(2.8)	0(0)	
Phase I/II	3(2.8)	1(1.4)	2(5.4)	
Phase II/III	3(2.8)	1(1.4)	2(5.4)	
Phase I/II/III	1(0.9)	1(1.4)	0(0)	
Unclear	27(25.0)	19(26.8)	8(21.6)	
**Area of disease**				0.28^a^
Oncology	28(25.9)	19(26.8)	9(24.3)	
Neurology	15(13.9)	10(14.1)	5(13.5)	
Infectious diseases	9(8.3)	8(11.3)	1(2.7)	
Respiratory	9(8.3)	3(4.2)	6(16.2)	
Hematology	8(7.4)	6(8.5)	2(5.4)	
Other	39(36.1)	25(35.2)	14(37.8)	
**Type of control**				0.56^a^
Non-active control	52(48.1)	32(45.1)	20(54.1)	
Active control	49(45.4)	35(49.3)	14(37.8)	
Both	7(6.5)	4(5.6)	3(8.1)	
**Type of outcome**				0.52
Clinical outcome	64(59.3)	40(56.3)	24(64.9)	
Surrogate endpoint	44(40.7)	31(43.7)	13(35.1)	
**Expected sample sizes** ^**b**^	162(86,400)	457(106, 423)	188(60,220)	< 0.01
**Randomized sample sizes** ^**b**^	124(69, 290)	141(89, 385)	86(42, 176)	< 0.01
**Funding**				
Private for profit	66(61.1)	41(57.7)	25(67.6)	0.43
Government	29(26.9)	25(35.2)	4(10.8)	0.01
Private not for profit	27(25.0)	21(29.6)	6(16.2)	0.20
Not funded	1(0.9)	1(1.4)	0(0)	1.00^a^
Unclear	14(13.0)	9(12.7)	5(13.5)	1.00

Values in parentheses are percentages unless indicated otherwise

^a^Fisher’s exact test

^b^Values are median (first, third quartile) and *P* value are from Wilcoxon rank-sum test

Adaptive trials published in JCR Q1 included more international trials than others (28.2% vs. 8.1%, *p* = 0.03). The clinical phase distributions differed between trials published in JCR Q1 and others (*p* = 0.01). Fewer JCR Q1 trials considered multiple adaptive designs than others (49.3% vs. 73.0%, *p* = 0.03). The expected and randomized sample sizes in JCR Q1 trials were larger than in others (median, 457 vs. 188, *p* < 0.01; 141 vs. 86, *p* < 0.01, respectively). The proportion of trials with governmental support was higher in the JCR Q1 trials than in others (35.2% vs. 10.8%, *p* = 0.01). Differences in other characteristics were not statistically significant.

### Adherence to the ACE checklist

Overall, of all the 26 topics in the ACE checklist, the adherence rate of the included trials ranged between 7.4% and 99.1%. Eight topics (30.8%) were reported adequately (adherence proportion ≥ 80%). “Interpretation” was the most adequately reported topic (*n* = 107, 99.1%), followed by “harms” (*n* = 103, 95.4%), and “numbers analyzed” (*n* = 100, 92.6%). Eight topics (30.8%) had poor adherence proportions (below 30%), including “SAP and other relevant documents” (*n* = 8, 7.4%), “blinding” (*n* = 12, 11.1%), “generalizability” (*n* = 17, 15.7%), “outcomes and estimation” (*n* = 23, 21.3%), “Implementation” (*n* = 24, 22.2%), “baseline data” (*n* = 25, 23.1%), “protocol” (*n* = 25, 23.1%), and “sequence generation” (*n* = 29, 26.9%) (Table 2 and Figure S3). A lower proportion of JCR Q1 trials adhered to the “baseline data” topic than other trials (14.1%, 40.5%, *p* < 0.01).

**Table 2:** Adherence to ACE checklist

Section	topic	Total (%) (*n* = 108)	JCR Q1(%) (*n* = 71)	Others (%) (*n* = 37)	*P* value
**Title and abstract**	1 Title and abstract	70(64.8)	50(70.4)	20(54.1)	0.14
**Introduction**	2 Background and objectives	95(88.0)	63(88.7)	32(86.5)	0.98
**Methods**	3 Trial design	92(85.2)	63(88.7)	29(78.4)	0.25
	4 Participants	78(72.2)	49(69.0)	29(78.4)	0.42
	5 Interventions	88(81.5)	58(81.7)	30(81.1)	1.00
	6 Outcomes	72(66.7)	49(69.0)	23(62.2)	0.62
	7 Sample size and operating characteristics	59(54.6)	40(56.3)	19(51.4)	0.77
**Randomization**	8 Sequence generation	29(26.9)	18(25.4)	11(29.7)	0.80
	9 Allocation concealment mechanism	34(31.5)	25(35.2)	9(24.3)	0.35
	10 Implementation	24(22.2)	17(23.9)	7(18.9)	0.72
	11 Blinding	12(11.1)	8(11.3)	4(10.8)	1.00
	12 Statistical methods	59(54.6)	40(56.3)	19(51.4)	0.77
**Results**	13 Participant flow	37(34.3)	22(31.0)	15(40.5)	0.44
	14 Recruitment and adaptations	65(60.2)	37(52.1)	28(75.7)	0.03
	15 Baseline data	25(23.1)	10(14.1)	15(40.5)	< 0.01
	16 Numbers analysed	100(92.6)	66(93.0)	34(91.9)	1.00
	17 Outcomes and estimation	23(21.3)	12(16.9)	11(29.7)	0.19
	18 Ancillary analyses	94(87.0)	63(88.7)	31(83.8)	0.67
	19 Harms	103(95.4)	67(94.4)	36(97.3)	0.84
**Discussion**	20 Limitations	34(31.5)	20(28.2)	14(37.8)	0.42
	21 Generalisability	17(15.7)	11(15.5)	6(16.2)	1.00
	22 Interpretation	107(99.1)	70(98.6)	37(100)	1.00^a^
**Other information**	23 Registration	65(60.2)	43(60.6)	22(59.5)	1.00
	24a Protocol	23(21.3)	18(25.4)	5(13.5)	0.24
	24b SAP and other relevant documents	8(7.4)	6(8.5)	2(5.4)	0.85
	25 Funding	92(85.2)	61(85.9)	31(83.8)	0.99

Values in parentheses are percentages unless indicated otherwise

^a^Fisher’s exact test

In terms of items specific to the adaptive design, only one of seven essential items (new items) was adequately reported. In addition, the adherence rate of the item was higher among JCR Q1 trials than among others (98.6% vs. 86.5%, *p* = 0.03). Three new items were underreported: 8 trials (7.4%) reported the SAP and other relevant documents, 14 (13.0%) studies mentioned measures to safeguard confidentiality of interim information and minimize potential operational bias during the trial, and 25 (23.1%) described assessments of similarity between interim stages. We found a statistically significant difference between JCR Q1 trials and others in terms of the similarity assessments (14.1% vs. 40.5%, *p* < 0.01). Of the remaining items, two targeted reporting of interim results and adaptive decisions made, and we found lower adherence rates among JCR Q1 trials than among others (17c item, 28.2% vs. 51.4%, *p* = 0.03; 14c item, 53.5% vs. 78.4%, *p* = 0.02) (Table 3).

**Table 3:** Adherence to adaptive design-specific items in ACE checklist

Topic	Item	Content of item (condensed)	Total (%) (*n* = 108)	JCR Q1 (%) (*n* = 71)	Others (%) (*n* = 37)	*P* value
**7 essential items (new items) for adaptive trials**						
**Trial design**	3b	Type of adaptive design used	102(94.4)	70(98.6)	32(86.5)	0.03
**Blinding**	11c	Measures to safeguard confidentiality and minimize operational bias	14(13.0)	10(14.1)	4(10.8)	0.86
**Statistical methods**	12b	statistical methods used to estimate treatment effects for adaptive design	77(71.3)	52(73.2)	25(67.6)	0.69
**Recruitment and adaptations**	14c	What trial adaptation decisions were made	67(62.0)	38(53.5)	29(78.4)	0.02
**Baseline data**	15b	Assessment of similarity between interim stages	25(23.1)	10(14.1)	15(40.5)	< 0.01
**Outcomes and estimation**	17c	Report interim results	39(36.1)	20(28.2)	19(51.4)	0.03
**SAP and other relevant trial document**	24b	Accessibility to full statistical analysis plan and other relevant trial documents	8(7.4)	6(8.5)	2(5.4)	0.85
**9 modified items for adaptive trials**						
**Trial design**	3c	No unplanned changes or unplanned changes with reasons	94(87.0)	61(85.9)	33(89.2)	0.86
**Outcomes**	6a	Define primary and secondary outcome and any other outcome for adaptive design	108(100)	71(100)	37(100)	1.00^a^
**Outcomes**	6b	No unplanned changes or unplanned changes with reasons	94(87.0)	62(87.3)	32(86.5)	1.00
**Sample size and operating characteristics**	7a	Determine sample size and operating characteristics	84(77.8)	57(80.3)	27(73.0)	0.53
**Sample size and operating characteristics**	7b	Pre-planned interim decision-making criteria; pre-planned and actual interim analysis	69(63.9)	45(63.4)	24(64.9)	1.00
**Sequence generation**	8b	Type of randomization; pre-planned and actual changes to allocation	37(34.3)	24(33.8)	13(35.1)	1.00
**Statistical methods**	12a	Statistical methods for primary and secondary outcomes and any other outcomes for adaptive design	71(65.7)	46(64.8)	25(67.6)	0.94
**Participant flow**	13a	Randomized and analysed number of participants	48(44.4)	29(40.8)	19(51.4)	0.40
**Recruitment and adaptations**	14a	Periods of recruitment and follow-up	73(67.6)	48(67.6)	25(67.6)	1.00
**6 items with expanded text for adaptive trials**						
**Recruitment and adaptations**	14b	Why the trial ended or was stopped	97(89.8)	61(85.9)	36(97.3)	0.13
**Baseline data**	15a	Baseline demographic and clinical characteristics	102(94.4)	66(93.0)	36(97.3)	0.62
**Numbers analysed**	16	Number of participants for analysis	100(92.6)	66(93.0)	34(91.9)	1.00
**Outcomes and estimation**	17a	Estimated effect size and its precision for primary and secondary outcome	58(53.7)	35(49.3)	23(62.2)	0.28
**Limitations**	20	Trial limitations, potential bias, imprecision, etc.	34(31.5)	20(28.2)	14(37.8)	0.42
**Generalisability**	21	Generalisability of the trial findings	17(15.7)	11(15.5)	6(16.2)	1.00

Values in parentheses are percentages unless indicated otherwise

^a^Fisher’s exact test

Of the nine modified items, three were adequately reported and none were underreported. Of the six items with expanded text, three were reported adequately and one for generalizability was reported poorly (*n* = 17(15.7%)) (Table 3). We found no statistically significant differences in either the modified or expanded items between JCR Q1 trials and others.

### Scores for the ACE checklist and potential factors associated with reporting quality

Based on our scoring strategy, the mean ACE checklist score of the 108 adaptive trials was 13.9 (SD, 3.5) out of 26, with 13.9 (SD, 3.5) in JCR Q1 trials and 14 (SD, 3.5) in other trials (*p* = 0.84). Both our univariable and multivariable regression analyses demonstrated that later published trials and the multicenter trials were associated with better reporting than the other trials (Table 4). We failed to find any associations between the type of outcome, the funding source, and the reporting quality.

**Table 4:** Univariable and multivariable analyses for reporting score

Study characteristics	Univariable analysis		Multivariable analysis	
	Coefficient	*P* value	Coefficient	*P* value
**Publication year**	0.14(0.05, 0.24)	< 0.01	0.14(0.05, 0.24)	< 0.01
**Trial center** **(multi-center vs. single-center)**	2.16(0.84, 3.48)	< 0.01	2.22(0.83, 3.61)	< 0.01
**Type of outcome** **(clinical outcome vs. surrogate endpoint)**	0.01(-1.36, 1.37)	0.99	0.20(-1.09, 1.49)	0.76
**Funding source** **(private for profit vs. other)**	0.60(-0.77, 1.97)	0.39	-0.35(-1.73, 1.04)	0.62

## Discussion

### Main findings and interpretations

We comprehensively identified the available adaptive drug RCTs and showed that the use of adaptive designs has been increasing. The adaptive designs have been applied mostly to speed the trials and facilitate decision-making. Adaptive designs have commonly been used in phase II and in oncology trials, with group sequential design being the most popular type. Adherence to the ACE checklist varied across 26 topics. We found adequate reporting for eight topics, and poor reporting for eight others. Moreover, we found a discrepancy between the new, modified, and expanded items, which are specific to adaptive designs in contrast to the CONSORT 2010 checklist. Through univariable and multivariable analyses, we explored potential influencing factors and found that trials published more recently and multicenter trials were associated with better reporting.

Our findings are partially consistent with those in other adaptive trial reviews [14–17, 26]. We found that adaptive designs have commonly been applied to phase II trials, whereas a previous review [17] that included phase II, phase III, and phase II/III RCTs on oncology found that adaptive designs were common in phase III trials. This discrepancy could be attributed to differing search strategies and inclusion criteria. Common applications in oncology and the frequent use of group sequential design were consistent findings in both our study and other reviews [14, 17]. The poor reporting identified in other studies [14–17] was limited to data monitoring, methodology, and accessibility to protocol. We also identified these deficiencies, which have been explicitly included as items or topics in the ACE checklist, such as the “measures for confidentiality” item and the “blinding” and “protocol” topics.

We also identified inadequate reporting of other important items, especially for those specific to the adaptive design. First, we consider the poor reporting of the “similarity assessment” item in the “baseline data” topic as a crucial matter. Baseline data may vary due to time drift, leading to dissimilarities between interim stages. This could affect analyses between and within different interim stages [27, 28], ultimately compromising the validity of the trials’ results. Second, the “outcomes and estimation” topic, which pertains to reporting of interim results, was insufficiently reported. This lack of reporting is not conducive to supporting interim decision-making. Unreasonable or unplanned adjustments may be made if actual decisions are contrary to what interim results direct, resulting in an increase in type I errors and incorrect conclusions [25]. Third, the reporting of the “SAP and other relevant documents” topic, a new topic added to ACE checklist, was unsatisfactory. Supporting documents could provide detailed information on adaptive designs, including the adaptive design type, statistical methods, and pre-planned decision-making criteria [29, 30], and these would increase the transparency and credibility of adaptive design trials. All of the above issues are critical for adaptive trials and should be taken seriously when reporting.

We identified additional general deficiencies that have also been prevalent in traditional trials, such as a failure to report allocation concealment and implementation. Moreover, we found that the more recently published and the multicenter trials were associated with more adequate reporting than other trials. This may reflect the widespread use of adaptive design and the increasing emphasis on the importance of its adequate reporting [31]. The rigorous quality control in multicenter trials had a significant role in this improvement [32].

### Strengths and limitations

Our study has several strengths. First, we included all phases of adaptive randomized trials and exposed their comprehensive characteristics. Second, we thoroughly assessed the reporting quality of the adaptive trials, using new, modified, and expanded items of the ACE checklist specifically tailored to adaptive trials. Third, we rigorously implemented a study process, which included developing inclusion and exclusion criteria, screening the literature, extracting data, and assessing report quality.

We are aware of the limitations of our review. First, our literature retrieval included only trials that explicitly claimed to be adaptive or used certain types of adaptive design. We failed to include trials with similar design details which did not explicitly claim an adaptive design. Therefore, it is possible that we missed some adaptive trials due to the limitations of our search strategy. Second, many topics contain multiple items, and our overall adherence rates for such topics do not accurately reflect adherence to each individual item. Finally, we only searched the literature up until 2020 to coincide with the publication of the ACE checklist. Hence, our results on reporting quality only exposed gaps in adaptive trials prior to the publication of the ACE checklist, highlighting areas for further improvement.

### Suggestions for reporting of drug adaptive randomized trials

Flexibility is a significant strength of adaptive designs, but it emphasizes the need for rigorous reporting of both pre-planned and actual changes in adaptive trials. Our results indicate that reporting on drug adaptive randomized trials is frequently inadequate, especially on essential items that include the SAP accessibility, confidentiality measures, and assessments of similarity between interim stages. This inadequate reporting may lead to ambiguity regarding planned modifications and the reasoning behind actual decisions, ultimately undermining the credibility of the findings from drug adaptive design trials.

Future adaptive trials should adhere to the ACE checklist to ensure that all pertinent details get reported, particularly regarding items essential to the adaptive design. Journals should consider requiring authors to follow the ACE checklist when reporting the design, analysis, and results of adaptive trials.

## Conclusion

The use of adaptive design has increased, and is primarily in early phase drug trials. Group sequential design is the most frequently applied method, followed by adaptive randomization, and adaptive dose-finding designs. However, the reporting quality of adaptive trials is suboptimal, especially in terms of essential items. Our findings suggest that clinical researchers need to provide adequate details of adaptive design and adhere strictly to the ACE checklist. Journals should consider requiring such information for adaptive trials.

### Electronic supplementary material

Below is the link to the electronic supplementary material.

Supplementary Material 1

## Data Availability

All data generated or analyzed during this study are included in this manuscript and supplementary files.

## Abbreviations

RCT: Randomized Controlled Trial
DMC: Dependent Monitoring Committee
CONSORT: Consolidated Standards of Reporting Trials
ACE: Adaptive designs CONSORT Extension
CENTRAL: Cochrane Central Register of Controlled Trials
JCR: Journal Citation Report
SD: Standard Deviation
Q1: Quantile 1
SAP: Statistical Analysis Plan
