# Supplementary material for: Adaptive designs were primarily used but inadequately reported in early phase drug trials
Source: BMC Med Res Methodol. 2024 Jun 5;24:130. doi: 10.1186/s12874-024-02256-9 (PMC11151552; doi:10.1186/s12874-024-02256-9)
Supplement: Supplementary file 1 — Supplementary Material 1 [file 12874_2024_2256_MOESM1_ESM.docx]

**Supplementary Material**

Records screened

(n=3597)

Duplicate records (n=1294)

Records excluded after title and abstract screening (n=3256）

Full-text articles assessed for eligibility

(n=341)

Exclude reports (n=234)

- Protocols (n=63)
- Trials that did not apply adaptive design (n=56)
- Methodological articles (n=46)
- Incomplete trials (n=23)
- Type of intervention was not drug (n=25)
- Non randomized trials (n=21)

107 records were included,

including 108 adaptive RCTs.

- JCR Q1 (n=71)
- Others (n=37)

Records identified through MEDLINE, EMBASE, CNETRAL, ClinicalTrials.gov database searching

(n=4891)

**Figure S1. Flowchart of article selection**


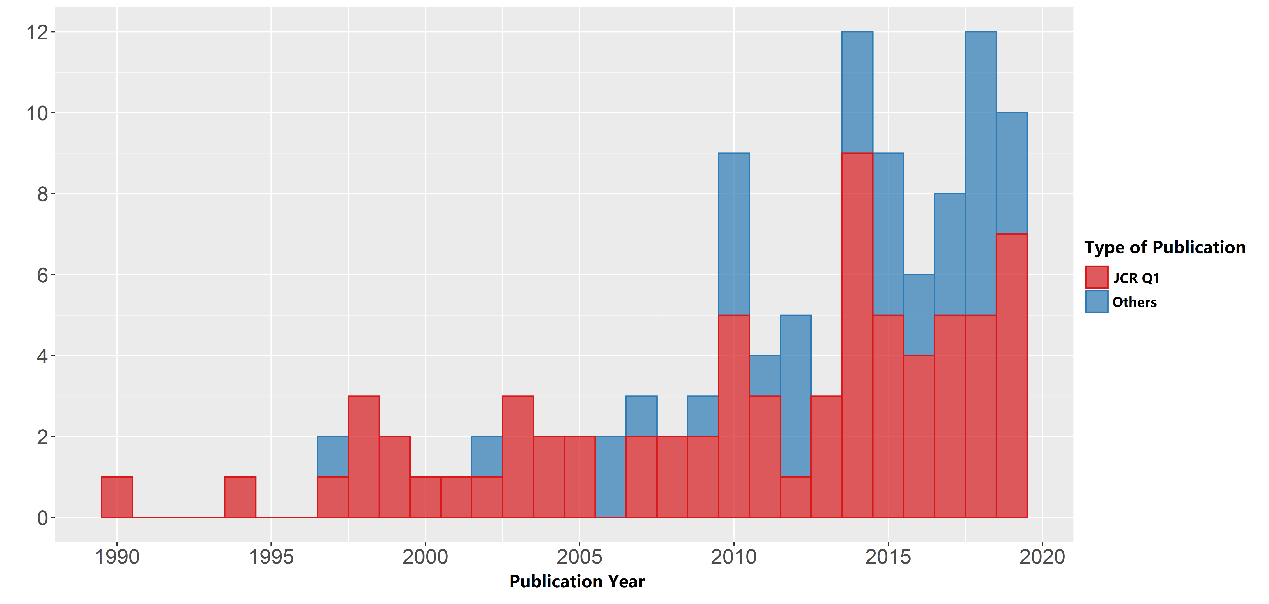


**Figure S2. Temporal trends in adaptive trials**

**Figure S3. Proportion of adherence to ACE checklist**

**Appendix 1. Search strategy**

**Searched through EMBASE on 2 January 2020**

1. Adaptive Clinical Trial/
2. Adaptive trial.mp.
3. Adaptive Design*.mp.
4. Adaptive randomization.mp
5. Adaptive seamless.mp
6. Adaptive hypothesis.mp.
7. Adaptive dose-finding.mp.
8. Biomarker adaptive.mp.
9. Biomarker adjusted.mp.
10. Group sequential.mp
11. Seamless design*.mp.
12. Treatment switching.mp.
13. Drop-the-loser.af.
14. Pick-the-winner.af.
15. Sample size re-estimation.mp.
16. Re-estimations.mp
17. Restricted randomization.mp
18. Or/1-17
19. "clinical trial (topic)"/
20. 18 and 19
21. 18 not 20
22. Review.pt
23. 21 and 22
24. 21 not 23
25. limit 24 to human

**Searched through MEDLINE on 2 January 2020**

1. Adaptive Clinical Trial/
2. Adaptive trial.mp.
3. Adaptive Design*.mp.
4. Adaptive randomization.mp
5. Adaptive seamless.mp
6. Adaptive hypothesis.mp.
7. Adaptive dose-finding.mp.
8. Biomarker adaptive.mp.
9. Biomarker adjusted.mp.
10. Group sequential.mp
11. Seamless design*.mp.
12. Treatment switching.mp.
13. Drop-the-loser.af.
14. Pick-the-winner.af.
15. Sample size re-estimation.mp.
16. Re-estimations.mp
17. Restricted randomization.mp
18. Or/1-17
19. Clinical Trials as Topic/
20. 18 and 19
21. 18 not 20
22. Review.pt
23. 21 and 22
24. 21 not 23
25. limit 24 to human

**Searched through CENTRAL on 2 January 2020**

1. Adaptive Clinical Trial/
2. Adaptive trial.mp.
3. Adaptive Design*.mp.
4. Adaptive randomization.mp
5. Adaptive seamless.mp
6. Adaptive hypothesis.mp.
7. Adaptive dose-finding.mp.
8. Biomarker adaptive.mp.
9. Biomarker adjusted.mp.
10. Group sequential.mp
11. Seamless design*.mp.
12. Treatment switching.mp.
13. Drop-the-loser
14. Pick-the-winner
15. Sample size re-estimation.mp.
16. Re-estimations.mp
17. Restricted randomization.mp
18. Or/1-17
19. Clinical Trials as Topic/
20. 18 and 19
21. 18 not 20
22. Review.pt
23. 21 and 22
24. 21 not 23
25. limit 24 to human

**Searched through clinical trial.gov on 2 January 2020**

Adaptive Clinical Trial or Adaptive trial or Adaptive Design or Adaptive randomization or Adaptive seamless or Adaptive hypothesis or Adaptive dose-finding or Biomarker adaptive or Biomarker adjusted or Group sequential or Seamless design or Treatment switching or Drop-the-loser or Pick-the-winner or Sample size re-estimation or Re-estimations or Restricted randomization
